# A case of post‐renal acute kidney injury caused by bilateral ureterolithiasis

**DOI:** 10.1002/ccr3.8825

**Published:** 2024-05-12

**Authors:** Junki Morino, Keiji Hirai, Yoshiyuki Morishita

**Affiliations:** ^1^ Division of Nephrology, First Department of Integrated Medicine Saitama Medical Center, Jichi Medical University Saitama Japan

**Keywords:** bilateral hydronephrosis, bilateral ureterolithiasis, post‐renal acute kidney injury, ureteric stent

## Abstract

**Key Clinical Message:**

Bilateral ureterolithiasis is rare but can cause acute kidney injury (AKI). Clinicians should first examine for post‐renal causes of AKI, even if the patient lacks subjective symptoms.

**Abstract:**

This letter describes a case of bilateral ureterolithiasis which presented with post‐renal acute kidney injury (AKI) and was successfully treated by bilateral retrograde ureteric stenting. Clinicians should be aware of post‐renal AKI caused by bilateral ureterolithiasis when acute worsening of renal function with oliguria is observed.

## 1 INTRODUCTION

Bilateral ureterolithiasis is a rare condition but it can cause serious complications such as post‐renal acute kidney injury (AKI). We report a case of bilateral ureterolithiasis which presented with post‐renal AKI and was successfully treated by bilateral retrograde ureteric stenting.

## 2 CASE REPORT

**FIGURE 1:**
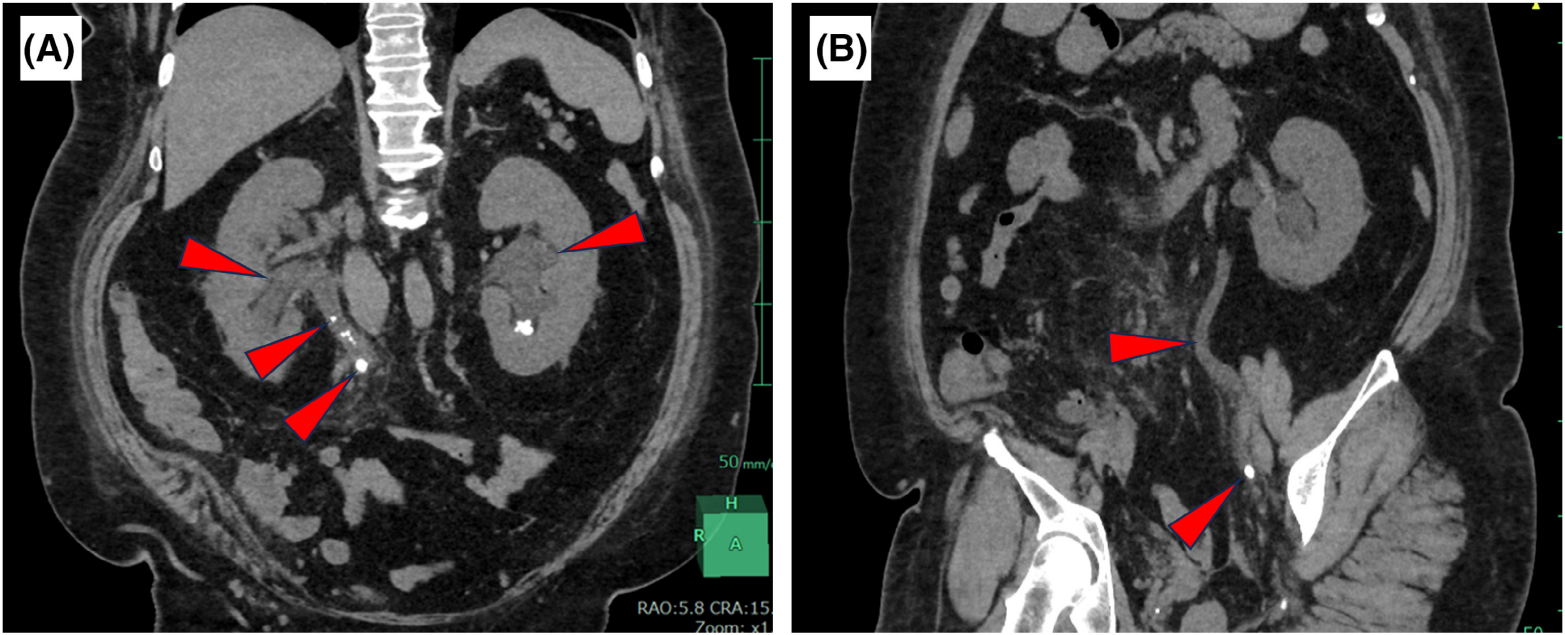
Computed tomography. (A) Coronal view showing bilateral hydronephrosis and right hydroureter and ureterolithiasis (red arrowheads). (B) Coronal view showing left hydroureter and ureterolithiasis (red arrowheads).

**FIGURE 2:**
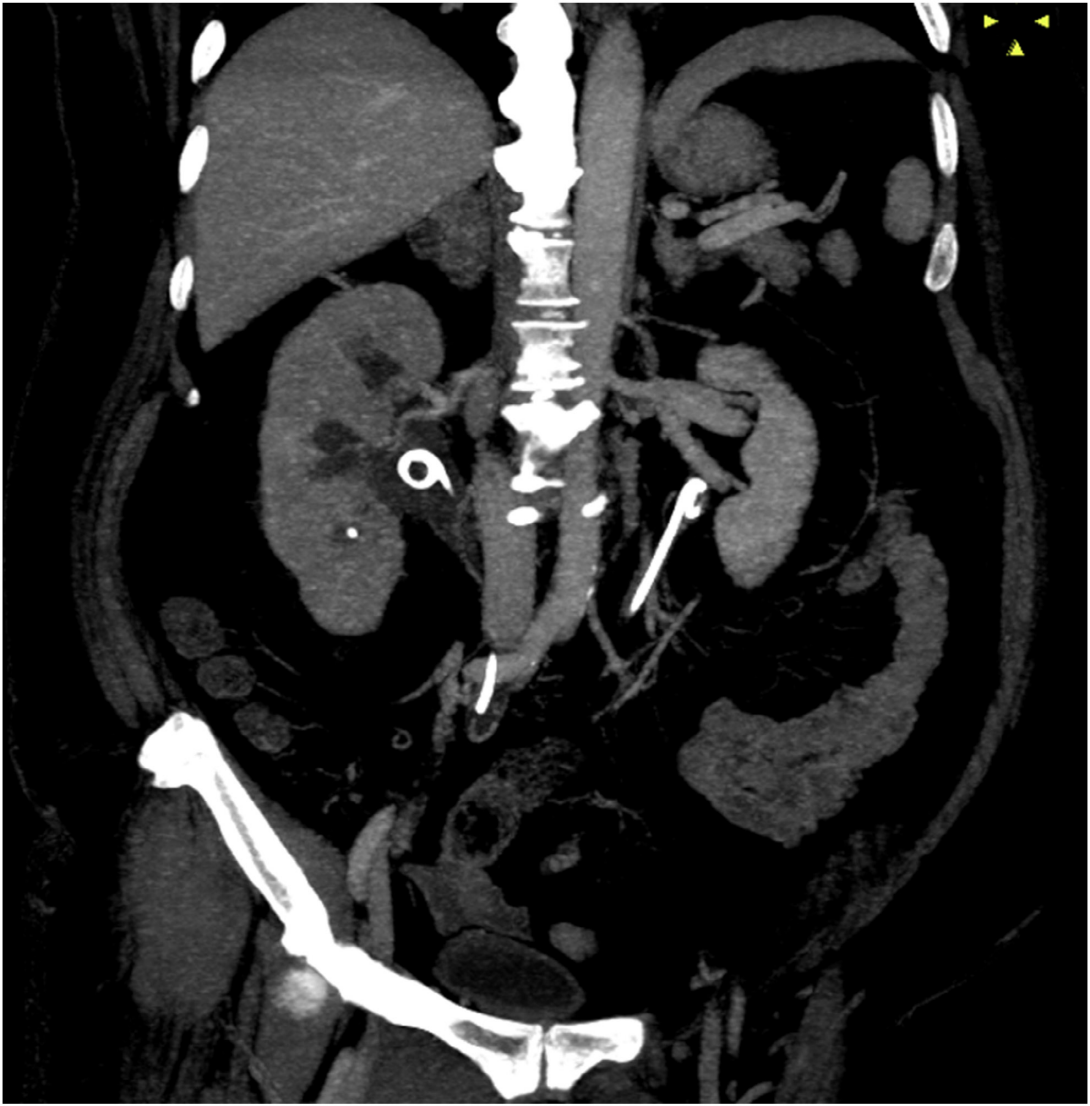
Computed tomography, coronal view, showing two double‐J stents in the right and left renal pelvis.

A 54‐year‐old woman was referred to our department with a 1‐week history of low urine output and acute worsening of renal function. She had no remarkable medical history except for atrial fibrillation treated with radiofrequency ablation 2 years previously. Physical examination showed no abdominal or costovertebral angle tenderness and stomachache. On admission, her vital signs were as follows: blood pressure, 180/103 mmHg; heart rate, 77 beats per minute; oxygen saturation, 96% on room air; and body temperature, 37.3°C. Laboratory investigations showed severe renal dysfunction (serum creatinine, 15.6 mg/dL (reference range, 0.46–0.82); blood urea nitrogen, 108 mg/dL (8.0–20.0)). Abdominal computed tomography showed bilateral hydronephrosis and ureterolithiasis (Figure 1). These stones were considered uric acid stones because she had hyperuricemia (15.3 mg/dL), obesity (body mass index 42.8 kg/m^2^), and aciduria (pH 5.0); and the stones were x‐ray‐negative and their CT value was 408 HU.^1^ On hospital day 2, double‐J stents were inserted bilaterally, leading to a remarkable increase in urine output from 100 mL/day before insertion to 2000–5000 mL/day after insertion (Figure 2). Serum creatinine improved progressively to 3.02 mg/dL on Day 4 and to 1.55 mg/dL on Day 7. She was discharged on Day 7 of hospitalization. At the 4‐week follow‐up examination, her serum creatinine level had decreased further to 0.91 mg/dL. She is scheduled to undergo transurethral lithotomy after complete recovery from AKI.

## 3 DISCUSSION

The common causes of post‐renal AKI include prostatic hypertrophy, pelvic malignancy, and inflammatory aortic aneurysms.^2^ Bilateral ureterolithiasis is rare but can cause post‐renal AKI. According to a previous report, only 5 of 2073 patients with ureterolithiasis developed AKI caused by bilateral ureterolithiasis.^3^ Ureterolithiasis often presents with clinical symptoms, such as flank pain, nausea, and hematuria^4^; however, in our case, there was no symptom except for oliguria before the diagnosis. Emergent surgical intervention is recommended in patients with bilateral ureterolithiasis because the condition results in AKI and persistent renal dysfunction.^5^ In this case, the patient's renal function rapidly improved to normal after bilateral retrograde ureteric stenting. Clinicians should be aware of post‐renal AKI caused by bilateral ureterolithiasis when acute worsening of renal function with oliguria is observed.

## AUTHOR CONTRIBUTIONS

**Junki Morino:** Writing – original draft. **Keiji Hirai:** Writing – review and editing. **Yoshiyuki Morishita:** Supervision.

## FUNDING INFORMATION

None.

## CONFLICT OF INTEREST STATEMENT

The authors have declared that no conflict of interest exists.

## ETHICS STATEMENT

All procedures performed in this study were in accordance with the ethical standards of the institutional and/or national research committee and with the 1964 Helsinki Declaration and its later amendments or comparable ethical standards.

## CONSENT

Written informed consent was obtained from the patient to publish this report in accordance with the journal's patient consent policy.

## Data Availability

Relevant data are available in the manuscript.
